# BMC Dermatology reviewer acknowledgement 2013

**DOI:** 10.1186/1471-5945-14-2

**Published:** 2014-03-20

**Authors:** Hayley Henderson

**Affiliations:** 1 BioMed Central, Floor 6, 236 Gray’s Inn Road, London WC1X 8HB, UK

## Abstract

**Contributing reviewers:**

The editors of BMC Dermatology would like to thank all our reviewers who have contributed to the journal in Volume 13 (2013).

## 

Mohamed Badawy Abdel-Naser

Egypt

Ayadi Ali

Tunisia

Tarek Amin

Saudi Arabia

Giuseppe Argenziano

Italy

Ioannis Bassukas

Greece

Carlos Brites

Brazil

Jorge Casseb

Brazil

Hector Chinoy

UK

Eung Ho Choi

Korea, South

John Clark

USA

Elliot Coups

USA

Soheil Dadras

USA

Thomas Dawson

Singapore

Remy Durand

France

Mahe Emmanuel

France

Steven Feldman

USA

Masaki Futamura

UK

Georgios Gaitanis

Greece

Stamatis Gregoriou

Greece

Shasa Hu

USA

Maria Celia Hughes

Australia

Kristina Ibler

Denmark

Hisayoshi Imanishi

Japan

Monika Janda

Australia

Ju-Hong Jeon

Korea, South

Neerja Jindal

India

Emina Kasumagic-Halilovic

Bosnia and Herzegovina

Tamihiro Kawakami

Japan

Leon Kircik

USA

Annett Korner

Canada

Ichiro Kurokawa

Japan

Chembolli Lakshmi

India

Cheng-Che Lan

Taiwan

Bodo Melnik

Germany

Nicolas Meyer

France

Esther Middelkoop

Netherlands

Anisa Mosam

South Africa

Kishwer Nehal

USA

David Nelson

USA

Ryuhei Okuyama

Japan

Aldona Pietrzak

Poland

Martial Piotto

France

Andor Pivarcsi

Sweden

June Robinson

USA

Julia Schofield

UK

Maria Do Rosario Silva

Brazil

Andrzej Slominski

USA

H. Peter Soyer

Australia

Doris Staab

Germany

Anthony Staines

Ireland

Cheila Stopiglia

Brazil

Mayte Suarez-Farinas

USA

Virginia Sybert

USA

Brian Tait

Australia

Kim Thomas

UK

Ariel Toloza

Argentina

Kursad Turksen

Canada

Uwe Wollina

Germany

Hisao Yamamura

Japan

Xianyong Yin

China

Philippa Youl

Australia

Jianzhong Zhang

China

Min Zheng

China

Kamiar Zomorodian

Iran

Christos Zouboulis

Germany

## Pre-publication history

The pre-publication history for this paper can be accessed here:

http://www.biomedcentral.com/1471-5945/14/2/prepub

